# Bone metabolism in diabetes: a clinician’s guide to understanding the bone–glucose interplay

**DOI:** 10.1007/s00125-024-06172-x

**Published:** 2024-05-18

**Authors:** Angela Sheu, Christopher P. White, Jacqueline R. Center

**Affiliations:** 1 Skeletal Diseases Program, Garvan Institute of Medical Research, Sydney, Australia; 2 Clinical School, St Vincent’s Hospital, Faculty of Medicine, University of New South Wales Sydney, Sydney, Australia; 3 Department of Endocrinology and Diabetes, St Vincent’s Hospital, Sydney, Australia; 4 Clinical School, Prince of Wales Hospital, Faculty of Medicine, University of New South Wales Sydney, Sydney, Australia; 5 Department of Endocrinology and Metabolism, Prince of Wales Hospital, Sydney, Australia

**Keywords:** Bone, Diabetes, Fractures, Insulin resistance, Osteoporosis, Review

## Abstract

Skeletal fragility is an increasingly recognised, but poorly understood, complication of both type 1 and type 2 diabetes. Fracture risk varies according to skeletal site and diabetes-related characteristics. Post-fracture outcomes, including mortality risk, are worse in those with diabetes, placing these people at significant risk. Each fracture therefore represents a sentinel event that warrants targeted management. However, diabetes is a very heterogeneous condition with complex interactions between multiple co-existing, and highly correlated, factors that preclude a clear assessment of the independent clinical markers and pathophysiological drivers for diabetic osteopathy. Additionally, fracture risk calculators and routinely used clinical bone measurements generally underestimate fracture risk in people with diabetes. In the absence of dedicated prospective studies including detailed bone and metabolic characteristics, optimal management centres around selecting treatments that minimise skeletal and metabolic harm. This review summarises the clinical landscape of diabetic osteopathy and outlines the interplay between metabolic and skeletal health. The underlying pathophysiology of skeletal fragility in diabetes and a rationale for considering a diabetes-based paradigm in assessing and managing diabetic bone disease will be discussed.

**Graphical Abstract:**

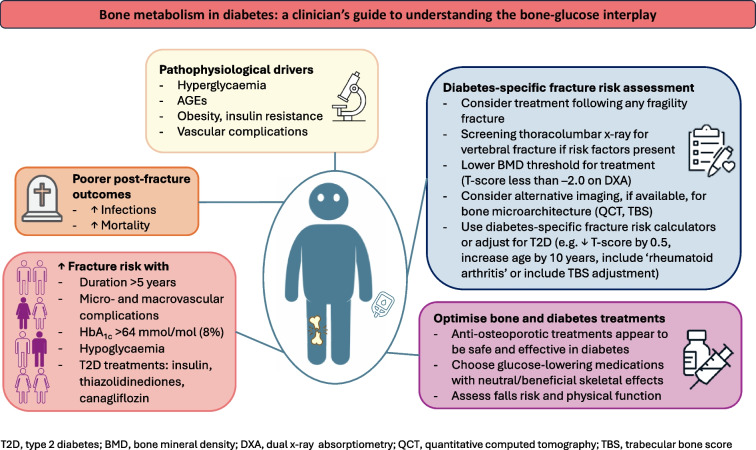

**Supplementary Information:**

The online version contains a slideset of the figures for download, which is available to authorised users at 10.1007/s00125-024-06172-x.


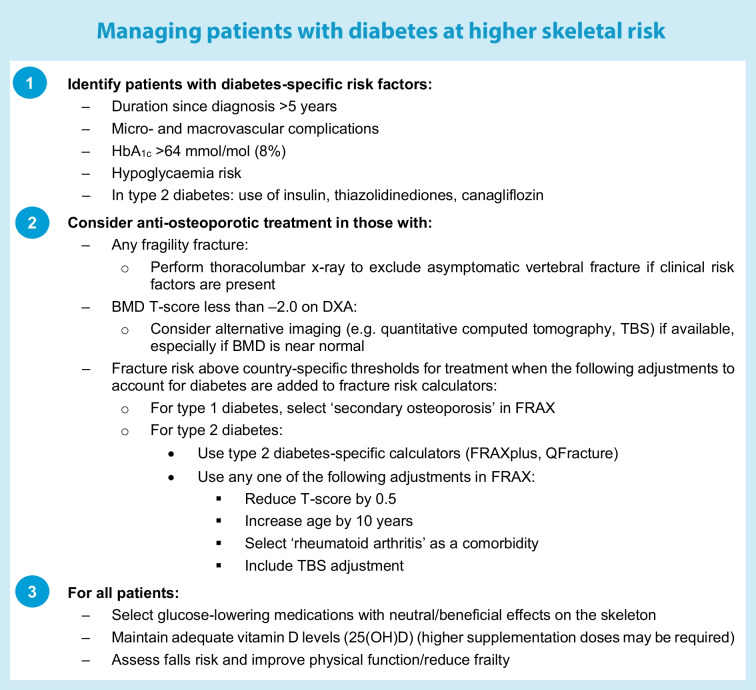


## Introduction

Skeletal fragility is increasingly being recognised as a complication of both type 1 and type 2 diabetes. Clinical studies are difficult to perform because of the heterogeneity of diabetic osteopathy and the lack of detailed concurrent bone and metabolic evaluation within study participants. However, risk of fracture and post-fracture mortality appear to be elevated in people with diabetes. Using a bone-centric framework for assessing skeletal health in diabetes has limitations; bone mineral density (BMD) and fracture risk calculators underestimate fracture risk in people with diabetes. The metabolic contributors to bone are multifactorial and complex, with many overlapping and contradictory effects of hyperglycaemia, hyperinsulinaemia and obesity on bone cells, structure and vasculature. Moreover, diabetes-related factors, including diabetes duration, glucose management, vascular complications and medications, may be specifically associated with bone deficits and fracture risk. Using a diabetes-centric approach to assessing bone may be more informative and may provide a framework for optimising the management of individuals with diabetes and skeletal fragility.

## Burden of skeletal fragility in diabetes

### Fracture risk in diabetes

Meta-analyses have shown an increased risk of any fracture in both type 1 and type 2 diabetes compared with no diabetes, with type 1 diabetes associated with the highest risk [1–4]. In type 1 diabetes, fracture risk is particularly elevated for hip fractures (RR 6.3–6.7), although the risk is also elevated for vertebral (RR 1.5–2.9) and non-vertebral (RR 3.3) fractures [1, 3, 4].

There is conflicting data for type 2 diabetes. Meta-analyses show an elevated risk of any fracture compared with no diabetes (RR 1.2), especially at the hip (RR 1.3–2.1) [2, 3]. However, in individual studies, the results are less consistent. Hip fractures are increased in some but not all studies [5, 6]. Increased risk may occur only in subsets of individuals with type 2 diabetes, including insulin users [5, 7], those with either a short [5, 8] or longer [5] duration of type 2 diabetes diagnosis or those with HbA_1c_ levels <53 mmol/mol (7%) [9] or >75 mmol/mol (9%) [10]. Studies examining fractures at non-hip sites are even fewer. Fractures of the ribs, humerus and distal leg/ankle are more common in type 2 diabetes [11, 12]. Wrist fractures may not be increased in type 2 diabetes [11, 12], despite the findings of a meta-analysis [3], which could have been driven by one case–control study. The risk of vertebral fractures also appears to be elevated in individuals with type 2 diabetes [11, 13], although some studies found no difference when compared with those without diabetes [2, 3], particularly in men [14]. In the most recent meta-analysis [13], type 2 diabetes was associated with an increased risk of vertebral fractures (OR 1.55, 95% CI 1.04, 2.31). Any (incident or prevalent) vertebral fracture was associated with increased risk of non-vertebral fractures and mortality. Despite the overall significant finding of increased risk of vertebral fractures in those with type 2 diabetes compared with those without in the pooled analysis, there was no difference in risk in the studies including individual participant data (five of 11 studies). This discrepancy could be due to ascertainment of vertebral fractures; as individuals with type 2 diabetes are more likely to have imaging for other clinical reasons, studies that do not use systematic radiographic examination for detecting vertebral fractures could underestimate vertebral fracture risk in those without type 2 diabetes and hence overestimate risk in those with type 2 diabetes. There was also significant loss to follow-up in the five population-based cohorts, which may have led to underestimation of incident vertebral fracture risk, particularly in the general population. These data suggest that routine spinal radiography in type 2 diabetes may be warranted, given both the high rates of otherwise undetected vertebral fractures and the adverse associations with vertebral fracture, which should prompt active management of bone health.

Fracture risk in diabetes, especially type 2 diabetes, is therefore not uniform and varies according to skeletal site. As there are no dedicated prospective studies evaluating fracture risk in diabetes, clinical characterisation of participants is limited and fracture ascertainment (methodology and skeletal sites) is restricted according to a study’s primary outcome. For example, hip fractures have been examined most frequently due to their ease of ascertainment through multiple study sources including linked database/registry studies. However, most fractures occur at peripheral sites and are associated with distinct risk factors (e.g. obesity and younger age) that may be of particular relevance to people with diabetes. Hence, adequately capturing fractures at all skeletal sites in individuals with and without diabetes is crucial to understanding the impact of diabetes on fracture risk.

Additionally, diabetes-related clinical characteristics affect fracture risk and thus defining a study cohort is essential for understanding the impact of diabetes on the skeleton. In the Dubbo Osteoporosis Epidemiology Study (DOES), type 2 diabetes (median type 2 diabetes duration of 6.3 years, 17% requiring insulin therapy) was not associated with increased fracture risk at any site over a median of 13 years of follow-up [15]. Similarly, in a Swedish cohort study, when 580,127 participants with type 2 diabetes from the national diabetes register were matched 1:1 with population-based control participants, type 2 diabetes was associated with only a marginal increase in risk of any fracture (adjusted HR [aHR] 1.07, 95% CI 1.05, 1.08) or hip fracture (aHR 1.11, 95% CI 1.09, 1.14) [12]. The proportion of risk explained by type 2 diabetes was <0.1%. However, among those with type 2 diabetes, significant (>20%) risk was associated with low BMI (<25 kg/m^2^), long type 2 diabetes duration (≥15 years), insulin treatment and absence of physical activity. Thus, the minimal increase in fracture risk overall was attributed to this cohort having relatively mild type 2 diabetes (55% of the cohort did not have any of the four risk factors), similar to the DOES study. Together, these findings highlight the heterogeneity of skeletal fragility and fracture risk in type 2 diabetes, and thus the importance of characterising study cohorts for type 2 diabetes-related features to allow for accurate interpretation and generalisability of study findings.

### Post-fracture mortality risk in diabetes

Concerningly, post-fracture outcomes are worse in those with diabetes than in those without. There is a paucity of data regarding post-fracture outcomes in type 1 diabetes alone (owing to the low numbers of participants with type 1 diabetes/inability to distinguish participants with type 1 diabetes from those with type 2 diabetes), with most studies grouping all participants with diabetes together. In a Taiwanese nested retrospective cohort study examining 30 day post-fracture outcomes, diabetes (3.1% with type 1 diabetes, 30.3% with type 2 diabetes) was associated with increased mortality risk, septicaemia, deep wound infection and urinary tract infection [16]. Among participants with diabetes, increased mortality risk was associated with higher glucose levels (OR 1.61) and type 1 diabetes (OR 1.93).

The already high mortality risk in the general population following hip fracture is further increased in type 2 diabetes in most studies [16–18]. Studies examining mortality following non-hip fractures are limited. Two studies found increased mortality risk in participants with type 2 diabetes following fractures at any skeletal site [15, 18]. In the DOES analysis, mortality risk following any fracture in type 2 diabetes was elevated (HR 2.62) over a median of 13 years [15]. The combination of fracture and type 2 diabetes conferred excess mortality risk greater than the sum of the individual risks, with post-fracture mortality (rather than type 2 diabetes-related mortality) driving the increased risk. Among those with type 2 diabetes, mortality risk was elevated even after non-hip non-vertebral (NHNV) fractures (HR 2.42), which is clinically significant given that more than half of fractures occur at NHNV sites. Longer duration of type 2 diabetes (>5 years) was associated with increased risk of mortality (HR 2.55–2.96, depending on fracture site) and there was a non-significant increase in mortality risk with insulin use (although numbers of participants were small).

The mechanisms driving increased risk of post-fracture mortality in diabetes are unclear. In the general population, fragility fractures at all sites are associated with substantial risk of mortality [19], and this risk varies according to skeletal site of fracture and comorbidities [20]. As type 2 diabetes is a chronic inflammatory condition associated with multiple end-organ complications and reduced functional status, exacerbation of these factors following a fracture, particularly in those with poorer premorbid function (e.g. type 2 diabetes with vascular complications or higher glucose levels), may contribute to premature mortality.

## Clinical features associated with skeletal fragility in diabetes

**Fig. 1:**
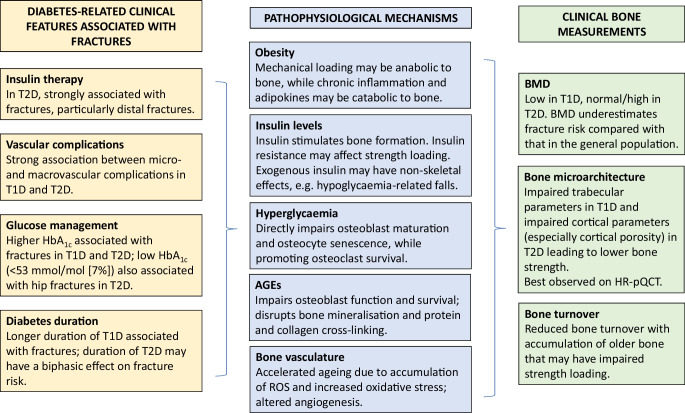
Summary of the clinical features and pathophysiology of skeletal fragility in diabetes. Numerous diabetes-related clinical characteristics are associated with increased fracture risk, although the independent contributors are difficult to ascertain because of significant clinical overlap. The contributing pathophysiological mechanisms are multifactorial, with many overlapping and sometimes conflicting effects. BMD is affected in diabetes (low in type 1 diabetes, normal/near-normal in type 2 diabetes) yet underestimates fracture risk compared with the general population for the same BMD level. Rather, impaired bone microarchitecture and low bone turnover result in impaired strength loading, suggesting a maladaptive response despite skeletal loading. HR-pQCT, high-resolution peripheral quantitative computed tomography; ROS, reactive oxygen species; T1D, type 1 diabetes; T2D, type 2 diabetes. This figure is available as part of a downloadable slideset

Diabetes-related clinical characteristics that are associated with increased fracture risk have been identified in epidemiology studies (Fig. 1). However, no prospective studies have been performed with the primary objective of establishing the diabetes-related predictors of fracture, and therefore the independent contributions of many inter-related features have been difficult to ascertain.

In type 1 diabetes, increased fracture risk is associated with microvascular complications, elevated HbA_1c_ levels, and longer type 1 diabetes duration [21]. Additionally, CVD is also associated with increased fracture risk, particularly in older people with long-standing type 1 diabetes [22].

Similarly, in type 2 diabetes, longer duration of type 2 diabetes [6, 12, 23], higher HbA_1c_ [6, 9, 10, 12, 24] and microvascular complications [6, 25] have all been associated with fracture risk. However, there have been conflicting studies, particularly around glucose levels, with an increase in hip fractures also observed in those with lower glucose levels (variably defined as HbA_1c_ from <48 to <53 mmol/mol [from <6.5% to <7%]) [9, 26]. A hypothesis for the J-curve relationship between glucose levels and fracture risk includes symptomatic hypoglycaemia contributing to falls. This is particularly pertinent as insulin therapy has been consistently shown to be associated with fractures, which could be related to hypoglycaemia [12, 27].

However, a significant limitation to understanding the diabetes-related contributors to fracture risk is the considerable co-occurrence of many of these features within one individual that cannot be adequately accounted for in non-prospectively collected studies. For example, people with type 2 diabetes with higher glucose levels are more likely to have vascular complications and require insulin therapy, and vascular complications (such as neuropathy and retinopathy) may be associated with falls. Studies designed with fracture endpoints typically do not include sufficient metabolic characterisation, thereby limiting the ability to adjust for confounding factors. In a unique post hoc analysis of the Fenofibrate Intervention and Event Lowering in Diabetes (FIELD) study, in which all on-study incident fractures were collected as part of the strict trial protocol and participants with type 2 diabetes were extensively characterised metabolically, we found independent associations between any fracture and macrovascular disease and HDL-cholesterol in men, between any fracture and neuropathy in women, and between any fracture and insulin therapy in both [28]. Although type 2 diabetes duration and baseline HbA_1c_ were associated with fractures in univariate analyses, they were no longer significant in the multivariable analyses, suggesting that these may be surrogate markers for more complicated type 2 diabetes that has not been fully adjusted for in other studies. Additionally, when proximal fractures (hip/vertebral and sites proximal to the elbow and knees) were separated from distal fractures, distal fractures were associated with microvascular disease and insulin therapy, while proximal fractures were mostly associated with age.

Together this study highlights several important key considerations. First, the association of fractures with vascular disease (independent of duration of disease or glucose levels) suggests that accelerated ageing and accumulation of AGEs, rather than cumulative hyperglycaemia per se, could drive skeletal fragility in the same way that they contribute to vascular complications. Second, the consistent association of insulin therapy with fractures, even after adjusting for confounders, suggests that insulin therapy itself (and not just complicated type 2 diabetes) is significant. As insulin is osteoanabolic, and insulin deficiency in type 1 diabetes affects peak bone mass, the contribution of insulin therapy may be through hypoglycaemia and falls. Finally, distinct risk profiles for proximal vs distal fractures suggest that distal fractures are particularly associated with diabetes-related factors, possibly reflecting an increased risk of falls, in contrast to the traditional osteoporosis-related risk factors for proximal fractures. Poorer functional status, including frailty [29], falls [24] and reduced/absence of physical activity [12], have all been associated with increased fracture risk in individuals with type 2 diabetes, highlighting the importance of considering non-skeletal factors for fracture risk. Further studies that account for the effects of hypoglycaemia, falls and physical performance on fractures at specific skeletal sites would be instructive.

## Pathophysiology of skeletal fragility in diabetes

The pathophysiology of skeletal fragility in diabetes is complex, with multiple co-existing, and often conflicting, contributors [30] (Fig. 1). Both type 1 and type 2 diabetes share commonalities of hyperglycaemia and vascular complications, both of which have direct and indirect detrimental effects on bone microarchitecture and structure. In contrast, differences in levels of endogenous insulin and the relative timing of skeletal maturation vs disease onset (diagnosis of type 1 diabetes at younger ages may precede accrual of peak bone mass) distinguish type 1 and type 2 diabetes and provide some insights into the relative mechanisms of metabolic effects on the skeleton.

### Hyperglycaemia

Direct cellular effects of hyperglycaemia include suppression of osteoblast maturation/differentiation, resulting in demineralisation of trabecular bone [31], and osteocyte senescence and accelerated apoptosis, which impairs mechanosensing and stress responses [32]. Despite fewer and smaller osteoclasts (from suppression of gene expression), chronic inflammation and higher fatty acids levels in the microenvironment promote their increased survival and greater resorption capacity, leading to imbalanced bone remodelling with bone resorption predominating over bone formation.

### Advanced glycation end-products

Development of AGEs following chronic hyperglycaemia is implicated in the pathogenesis of diabetes-related vascular complications through accelerated ageing. AGEs are associated with suppressed osteoblast development, function and survival, leading to lower bone formation and turnover [33]. Additionally, AGEs disrupt bone mineralisation and bone protein and collagen cross-linking, resulting in poorer microarchitecture and reduced capacity under stress loading [34]. In vitro assessment of AGEs is technically challenging; however, AGEs have been associated with features of ageing bone on iliac crest bone biopsy and levels correlate with HbA_1c_ and presence of vascular complications [35].

### Obesity and insulin resistance

Endogenous insulin stimulates hepatic expression of growth hormone and therefore IGF-1 production. Thus, insulin deficiency in type 1 diabetes is associated with decreased osteoblast stimulation and low bone formation [36]. In contrast, hyperinsulinaemic states, such as insulin resistance/early type 2 diabetes and congenital lipodystrophy, are associated with bone anabolism with high/normal BMD [37].

Mechanical loading from increased body weight in obesity is associated with higher BMD [38]. However, the contributions of metabolic effects, including chronic inflammation and adipokines, are less clear [30]. Proinflammatory cytokines stimulate osteoclast-driven bone resorption [39]. Leptin and adiponectin both appear to be anabolic to bone, although adiponectin may switch to predominantly catabolic effects in ageing and inflammatory states. Visceral adipose tissue (VAT) is the metabolically active tissue that is associated with adverse metabolic sequelae and is characterised by elevated levels of leptin and proinflammatory cytokines and lower levels of adiponectin. The data on the effect of VAT on the skeleton are conflicting but VAT appears to be positively associated with BMD, although the association reverses once BMI/body weight is accounted for [40]. Despite higher BMD and lower bone turnover, both VAT [38] and insulin resistance [41, 42] have been associated with inferior hip geometry and strength loading, suggesting that maladaptive skeletal loading despite preserved BMD may underpin the skeletal fragility observed in progressive type 2 diabetes.

### Vascular complications

As diabetes advances, accelerated ageing and associated vascular complications become increasingly pertinent in the pathogenesis of skeletal fragility. Both chronic hyperglycaemia and acute glycaemic fluctuations are associated with accumulation of reactive oxygen species and increased oxidative stress, leading to activation of pathways causing DNA and protein damage. These processes inhibit osteoblast differentiation and increase apoptosis in murine models of both type 1 and type 2 diabetes [43], providing a common mechanism linking the development of vascular complications and skeletal fragility. Similarly, vascular endothelial growth factor (VEGF), a key determinant of angiogenesis and hence diabetic complications (particularly proliferative diabetic retinopathy and nephropathy), plays a vital role in bone vascularisation, osteoblast differentiation and bone repair/regeneration.

### Secretory functions of bone that influence metabolism

Although limited, there is evidence that skeletal hormones may also influence glucose homeostasis.

Osteocalcin is secreted by osteoblasts, under stimulation by active vitamin D (1,25(OH)_2_D), and regulates both osteoblastic and osteoclastic activity and bone mineralisation [44]. Associations between osteocalcin and glucose management have been identified, although the direction of association remains unclear, particularly as findings in animal models have not been confirmed in human studies. In a series of mice models, osteocalcin deficiency was associated with decreased pancreatic beta cell proliferation, glucose intolerance and insulin resistance [45]. Positive associations between osteocalcin and insulin sensitivity have been observed in older men with [46] and without [47] type 2 diabetes. However, there is limited data on whether interventions that improve osteocalcin levels improve glucose metabolism, and there remains significant debate as to the metabolic significance of the carboxylated vs uncarboxylated forms of osteocalcin [44].

Osteoglycin is a proteoglycan that is expressed in many tissues, including bone and muscle [48]. There is conflicting data on the effects of osteoglycin on bone metabolism, with evidence of both osteoblastic inhibition and stimulation during osteoglycin overexpression in preclinical studies [48]. In a sophisticated study in osteoglycin-deficient mice, BMD and femur length were increased compared with wild-type mice, and this was shown to be related to increased osteoblast activity, increased mineralisation and decreased osteoclast numbers [49]. Additionally, osteoglycin-deficient mice had impaired glucose tolerance with evidence of insulin resistance, both of which improved following osteoglycin treatment. In a parallel study of humans with obesity undergoing weight loss interventions, post-intervention circulating osteoglycin levels were elevated, and levels were positively correlated with weight loss and change in BMI and negatively correlated with fasting glucose levels [49]. Together, this suggested a common mediator of bone and glucose/energy homeostasis, whereby osteoglycin regulates insulin sensitivity and facilitates skeletal adaptation during energy/weight change. However, two cross-sectional studies did not find any associations between osteoglycin levels and HbA_1c_ [50, 51]. Thus, the role of osteoglycin in modulating glucose metabolism requires further research.

## Skeletal assessment in diabetes

### Clinical bone assessment in diabetes

The ideal investigations to identify individuals with diabetes at elevated fracture risk remain unclear [52]. Both type 1 and type 2 diabetes are associated with changes in areal BMD (aBMD) when measured by dual-energy x-ray absorptiometry (DXA) (Fig. 1). In type 1 diabetes, BMD is low, probably because of inadequate accrual of peak bone mass due to hypoinsulinaemia and lower levels of IGF-1 [3, 53]. In a cross-sectional study of the long-term Epidemiology of Diabetes Interventions and Complications (EDIC) study, higher HbA_1c_ and nephropathy were independently associated with lower aBMD in older (59.2±6.7 years) participants with type 1 diabetes [54]. In contrast, aBMD is relatively preserved and even elevated in type 2 diabetes and is related to increased body size [38]. However, in both type 1 and type 2 diabetes, fracture risk is higher than predicted based on aBMD levels [3]. Nevertheless, low aBMD remains a predictor for fractures in type 2 diabetes [15].

Rather than deficits in BMD, diabetes may increase skeletal fragility through altered microarchitecture, including increased cortical porosity, and low bone turnover (Fig. 1). High-resolution peripheral quantitative computed tomography (HR-pQCT) provides in vivo assessment of volumetric BMD (vBMD) and trabecular/cortical compartments of the distal radius and tibia. As with most diabetes-related studies, individual studies are confounded by significant clinical heterogeneity in diverse cohorts. A recent meta-analysis found site-specific differences in bone structure between people with type 1 and type 2 diabetes and those without diabetes [55]. Compared with control participants without diabetes, type 1 diabetes was associated with impaired trabecular parameters (vBMD, number, and heterogeneity) at the radius but not the tibia. Cortical parameters were preserved. In contrast, type 2 diabetes was associated with preserved trabecular features and enhanced cortical thickness but increased cortical porosity (particularly at the radius). Conversely, in a recently published cohort of 59 older individuals with long-standing type 1 diabetes (duration 37.7±9.0 years, age 59.9±9.9 years), type 1 diabetes was associated with poorer cortical measurements (thickness, vBMD) at the ultradistal tibia but not the radius [56]. However, cortical changes (and decreased bone strength and stiffness) were dependent on the presence of diabetic neuropathy, suggesting that changes may have been driven by vascular complications. Similarly, in three studies of type 2 diabetes, cortical changes were not observed in all those with type 2 diabetes but only in those with previous fracture [57], microvascular complications [58] or clinically significant peripheral vascular disease [59].

Together, HQ-pQCT data provide several insights. First, differences between the radius and the tibia suggest that mechanical load, and therefore obesity, may affect bone microarchitecture. Second, differences between type 1 and type 2 diabetes, and the association of changes in HQ-pQCT with vascular complications, underscore the complex interplay of metabolic factors, vascular complications and age, especially as the phenotype of older type 1 diabetes appears to resemble that of type 2 diabetes. Further studies in well-characterised individuals with type 1 and type 2 diabetes and examining the role of HR-pQCT parameters in fracture risk prediction are warranted.

Bone turnover is best assessed by tetracycline-labelled iliac bone biopsy, although the invasiveness of this technique limits its widespread use in clinical practice and research studies. Histomorphometry studies have shown older bone with reduced bone turnover and abnormal collagen structure in insulin-requiring women with type 2 diabetes [60]. Changes were not associated with type 2 diabetes duration or HbA_1c_ levels. Type 2 diabetes has also been associated with stiffer and harder cortical indices and relatively preserved trabecular mechanical properties [35].

Serum bone turnover markers (BTMs) can be used to non-invasively assess bone turnover clinically. Although there are some conflicting studies, meta-analyses suggest BTMs reflecting bone formation and resorption are reduced in both type 1 diabetes [61] and type 2 diabetes [62]. Separating out the metabolic contributors to lower BTMs has been challenging, with inconsistent associations with HbA_1c_ [63], adiposity [64] and microvascular complications [65]. In our detailed cross-sectional analysis of the DOES cohort, type 2 diabetes was independently associated with lower BTMs (25–50% lower than in those without type 2 diabetes) [38]. Insulin resistance, but not obesity or visceral adiposity, was also associated with lower BTMs, suggesting that hyperinsulinaemia may be a key pathophysiological contributor. However, the utility of BTMs in fracture prediction in type 2 diabetes remains unclear, as one case–control study found that BTMs were directly associated with fracture risk in participants without type 2 diabetes, but not in those with type 2 diabetes [66], and prospective studies are required.

Given the limitations of conventionally derived aBMD using DXA in diabetes, other clinically available modalities such as trabecular bone score (TBS) and advanced hip analysis (AHA) are being investigated. The TBS indirectly measures lumbar spine trabecular microarchitecture by evaluating grey-level variations in pixels from a spine DXA image. The TBS is probably lower in individuals with type 1 diabetes than in those without type 1 diabetes [67], although there may be no differences in younger people (aged 19–50 years) with type 1 diabetes without diabetic complications [68]. Similarly, type 2 diabetes is associated with a lower TBS [69] and this has been shown to partially explain the fracture risk in type 2 diabetes [70]. The lower TBS in type 2 diabetes appears to be associated with BMI and fat mass [71], and therefore abdominal adiposity rather than type 2 diabetes per se may drive the apparent discrepancy of poorer trabecular bone on TBS compared with the preserved trabecular parameters seen in the HR-pQCT data.

AHA uses hip DXA geometry and structural parameters to estimate hip strength. In one study, type 1 diabetes was associated with poorer cortical measurements and femoral neck instability, although the participants in this study also had end-stage kidney disease [72]. Both type 2 diabetes and impaired glucose tolerance have been associated with worse strength parameters in some but not all studies, especially when adjusted for lean/total body mass [73, 74]. AHA parameters in type 2 diabetes appear to be associated with BMI and body size, rather than type 2 diabetes, although visceral adiposity is inversely associated with some measures of skeletal load strength [38]. Further characterisation of AHA changes across various dysglycaemic states would clarify its clinical utility.

### Fracture risk calculators

The combination of fractures and diabetes is associated with poor outcomes. However, as discussed, the clinical risk factors for fracture are not well established and current fracture risk calculators inadequately estimate fracture risk in diabetes [52]. Compared with the general population, neither aBMD nor the Fracture Risk Assessment Tool (FRAX; https://frax.shef.ac.uk/FRAX/index.aspx, accessed 30 April 2024) fully capture fracture risk in type 2 diabetes. The FRAX fracture risk in type 2 diabetes was found to be equivalent to that of an age- and sex-matched counterpart without type 2 diabetes with a T-score of 0.4–0.6 lower [75]. Adjusting the FRAX inputs with one of the following four factors improved fracture prediction in type 2 diabetes, but was still insufficient to fully explain the fracture risk: reduce T-score by 0.5, increase age by 10 years, include ‘rheumatoid arthritis’ as a comorbidity in place of type 2 diabetes, or add the TBS adjustment [76]. Type 1 diabetes can be adjusted for by selecting ‘secondary osteoporosis’ in FRAX, although it is one of six clinical conditions grouped together in this category. Type 2 diabetes has recently been added as an option for inclusion in the paid add-on beta version of FRAXplus (https://www.fraxplus.org/frax-plus, accessed 30 April 2024). Whether this improves identification of higher risk patients is unknown. QFracture (https://qfracture.org/, accessed 30 April 2024), derived from a UK prospective cohort of general practices, is the only freely available calculator that includes type 2 diabetes as a variable for fracture risk calculation. However, its widespread use is limited, particularly outside the UK, because of limited ascertainment of risk factors in the algorithm derivation.

Although accounting for type 2 diabetes as a clinical risk factor improves fracture risk prediction, including it as a dichotomous variable does not fully consider the clinical heterogeneity of diabetes or diabetes-related skeletal risk. Rather, diabetes-specific features should be used to adequately quantify fracture risk in diabetes. In the Fremantle Diabetes Study Phase I, a longitudinal observational diabetes cohort study with linked hip fracture hospitalisations, five clinical characteristics (older age, female sex, lower BMI, peripheral sensory neuropathy and reduced renal function) were identified as the significant predictors sufficient for calculating 10 year hip fracture risk [77]. Further studies on other skeletal sites and in type 1 diabetes are required.

There are no evidence-based guidelines on optimal assessment and management of bone health in diabetes, although two algorithms for type 2 diabetes have been proposed [78, 79]. Principles for managing patients with diabetes at higher skeletal risk are summarised in the text box (‘Managing patients with diabetes at higher skeletal risk’). Treatment thresholds should be adjusted in diabetes (e.g. T-score less than −2.0, fracture risk calculator adjustments) given that fracture risk is underestimated by aBMD when measured by DXA. We propose that fragility fractures at all sites should prompt treatment initiation given that post-fracture mortality risk is elevated following all fractures. Routine thoracolumbar x-ray screening is warranted, particularly in those at higher skeletal risk (either diabetes-related or general clinical risk factors). Currently available treatments appear to be effective and safe in type 2 diabetes, although there are no prospective trials investigating these agents specifically in type 2 diabetes (see the next section) and very limited data in type 1 diabetes. Bone anabolic therapies may be particularly advantageous in type 2 diabetes, but further data are required before a particular anti-osteoporotic medication is recommended over any other.

## Optimising management of skeletal fragility in type 2 diabetes

### Bone treatments in diabetes

**Fig. 2:**
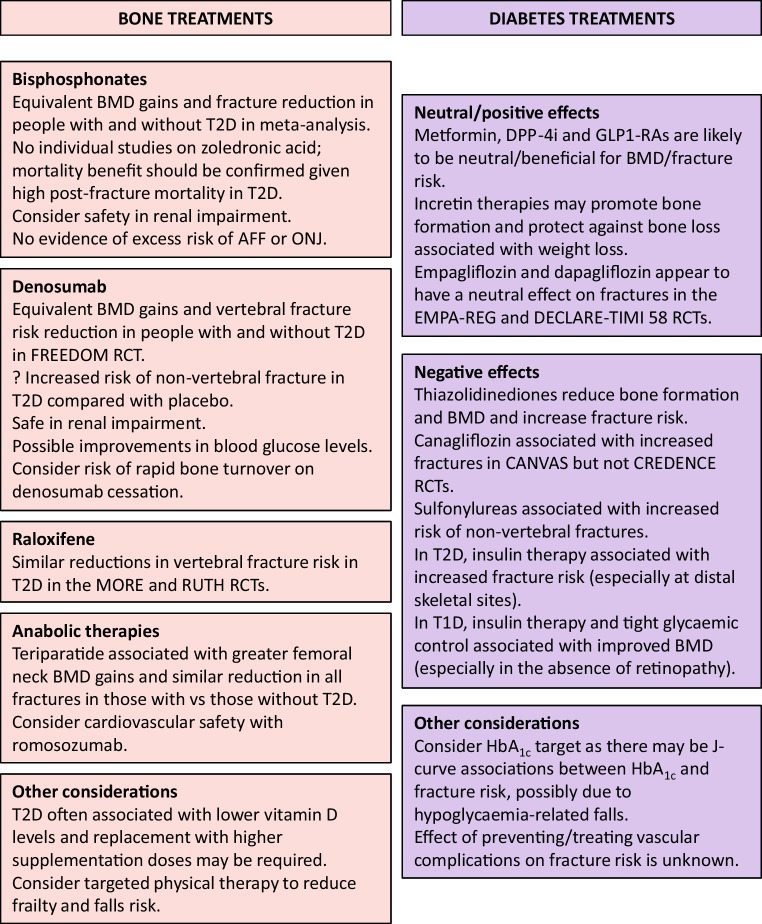
Optimising the management of skeletal fragility in diabetes. There are no prospective RCTs establishing the optimal management of people at risk of diabetic osteopathy. Post hoc analyses suggest that anti-osteoporotic treatments are probably at least as effective in type 2 diabetes as in the general population. Anabolic therapies may have additional benefits in type 2 diabetes given the underlying low bone turnover. The safety and efficacy of anti-osteoporotic medications in normal/near-normal BMD is unknown. With the increasing use of glucose-lowering medications for non-glycaemic benefits (including weight loss and cardiovascular and renal benefits), the effects on the skeleton need to be established and agents with neutral/positive bone effects considered in those at high skeletal risk. AFF, atypical femoral fracture; DPP-4i, dipeptidyl peptidase-4 inhibitors; GLP1-RA, glucagon-like peptide-1 receptor agonist; ONJ, osteonecrosis of the jaw; T1D, type 1 diabetes; T2D, type 2 diabetes. This figure is available as part of a downloadable slideset

The unique skeletal phenotype in diabetes raises questions about the optimal management of diabetic osteopathy [52] (Fig. 2). No prospective RCTs have evaluated the efficacy and safety of treatments for osteoporosis in people with diabetes. Post hoc analyses of trial data are limited to type 2 diabetes, given the small number of participants with type 1 diabetes. Compared with participants without type 2 diabetes, alendronate treatment resulted in similar BMD gains in those with type 2 diabetes in the placebo-controlled Fracture Intervention Trial [80] and equivalent fracture reduction in a prescription registry cohort [81]. Three risedronate trials showed equivalent BTM reductions and BMD improvements in those with and without type 2 diabetes [82]; there are no comparative data on the effect of risedronate on fracture risk. There are no individual studies of zoledronic acid in type 2 diabetes, but in a meta-analysis of 15 RCTs using antiresorptive agents (including two zoledronic acid RCTs), bisphosphonates were effective in improving BMD and reducing fracture risk [83]. Importantly, the mortality benefit of zoledronic acid following hip fracture [84] should be confirmed in people with type 2 diabetes specifically, given the high post-fracture mortality risk associated with type 2 diabetes. The use of bisphosphonates needs to be carefully considered in people with type 2 diabetes given its contraindication in renal impairment.

Raloxifene was associated with similar reductions in vertebral fracture risk in women with type 2 diabetes in post hoc subgroup analyses of theMORE [85] and RUTH [86] RCTs.

In the FREEDOM and extension trials using denosumab, BMD gains and vertebral fracture risk reduction were similar between participants with and without type 2 diabetes [87]. However, non-vertebral fracture risk appeared to be increased in type 2 diabetes with denosumab compared with placebo. The reasons for this finding are unclear, but it occurred only in the first 3 years and could have been related to lower than anticipated fracture numbers in the placebo-treated participants with diabetes. Further studies to confirm the effect on non-vertebral fractures in type 2 diabetes are warranted.

In addition to the advantage of being safe in renal impairment, denosumab may also have additional benefits in type 2 diabetes due to improvements in glycaemia. A single dose of denosumab was associated with improved HbA_1c_ [88, 89], although there were no changes in fasting glucose or insulin levels. In the FREEDOM trial, denosumab improved glucose levels only in participants with untreated type 2 diabetes [90]. Gene mapping studies suggest that denosumab suppresses *DPP4* gene function and this may be more clinically evident in those with established dysglycaemia [89]. Longer term effects have not been established and treatment for potential metabolic benefits should be considered alongside the risk of rapid bone turnover that occurs on medication cessation.

Given that type 2 diabetes is characterised by low bone turnover, anabolic therapies may be preferred. In the DANCE observational study, teriparatide resulted in greater improvements in femoral neck BMD, similar improvements in spine and total hip BMD, and similar reductions in non-vertebral fractures in those with type 2 diabetes compared with those without [91]. In a subsequent study that included three additional observational studies, teriparatide was associated with a greater reduction in all clinical fractures in those with type 2 diabetes compared with those without [92]. Participants with type 2 diabetes experienced comparable improvements in BMD and TBS with abaloparatide in the ACTIVE trial [93]. Preclinical studies suggest that treatment with teriparatide, abaloparatide and romosozumab increases bone formation, corrects cortical porosity and improves mechanical properties in a skeletally mature mouse model of diabetes [94]. However, romosozumab treatment in people with type 2 diabetes needs to be carefully considered given the increased risk of cardiovascular events in the alendronate-controlled ARCH trial [95].

There was no signal for increased risk of atypical femoral fractures or osteonecrosis of the jaw in participants with type 2 diabetes, although on-trial adverse events were rare overall across all drug RCTs. There are no data on the effect of these agents on bone microarchitecture in diabetes, nor on efficacy in those with normal/near-normal BMD.

### Effect of diabetes medications on the skeleton

Similarly, the effects of diabetes and obesity medications on the skeleton need to be established (Fig. 2). Data are limited to post hoc analyses, with significant limitations to the generalisability of some studies. With the significantly increased use of sodium–glucose cotransporter 2 inhibitors (SGLT2i) and incretin therapies in people with and without type 2 diabetes, the potential skeletal effects directly from the medication and from secondary metabolic changes need to be considered. Additionally, risk of hypoglycaemia and falls should be considered.

In type 1 diabetes, one study showed that 7 years of intensive insulin therapy improved BMD and decreased bone resorption markers [53]. Benefits were less marked in those with retinopathy, although the separate effects of duration of disease, glucose management, BMI and other vascular risk factors were not examined. Continuous insulin infusion in a murine model of type 1 diabetes led to a dose-dependent increase in bone formation markers and decrease in bone resorption markers, and improvements in femoral cortical and trabecular parameters and strength measurements, despite elevated glucose levels well above the non-diabetic level [96]. Clinical studies establishing the effect of insulin treatment and modification of vascular complications with respect to age of type 1 diabetes diagnosis and duration of disease would provide insights into the skeletal benefits beyond glycaemia.

In type 2 diabetes, use of metformin [97, 98], dipeptidyl peptidase-4 (DPP-4) inhibitors [99] and glucagon-like peptide-1 receptor agonists (GLP1-RAs) [100] appears to be neutral/beneficial with regard to BMD and fracture risk. Incretin therapies (DPP-4 inhibitors and GLP1-RAs) may directly promote bone formation and inhibit bone resorption [100]. Treatment with liraglutide prevented bone loss and increased bone formation marker levels following low energy diet-induced weight loss in women with obesity and without diabetes [101]. Studies of incretin therapies in type 2 diabetes would be particularly useful given the potential concurrent weight loss and non-glycaemic benefits.

SGLT2i are increasingly being used for their cardiovascular and renal benefits. However, concerns were raised in the landmark CANVAS RCT designed to investigate cardiovascular outcomes after canagliflozin treatment led to increased lower limb amputations and fractures [102]. However, there was no increase in fracture risk in the subsequent CREDENCE trial with primary renal endpoints [103], nor in the cardiovascular outcome trials of empagliflozin (EMPA-REG) [104] and dapagliflozin (DECLARE-TIMI 58) [105]. Pooled analyses have not found an effect on fracture risk of treatment with any SGLT2i [106, 107]. As fracture risk is greatest in those with vascular complications, and such individuals would benefit most from the non-glycaemic effects of these agents, it is important to establish whether fracture risk is limited to the clinical cohort of the CANVAS trial or whether there are specific drug/class effects.

Agents associated with increased fracture risk that should be used cautiously in people at risk for skeletal fragility include thiazolidinediones, sulfonylureas and insulin. The thiazolidinediones affect gene expression, leading to impaired osteoblast differentiation and reduced bone formation. They have been associated with reduced BMD and increased fracture risk, particularly in women [108]. Sulfonylureas have been associated with an increased risk of non-vertebral fractures [27] but not radiological vertebral fractures [14] in elderly men in the MrOS study. Given that non-vertebral fractures were associated with insulin users and falls, it was postulated that hypoglycaemia-related falls could be contributing to this observation.

In most studies, insulin therapy in type 2 diabetes is associated with increased fracture risk, with some studies finding the type 2 diabetes-related fracture risk only in insulin users [5–7]. Given the anabolic effects of insulin, the mechanism has been hypothesised to be related to the complexity of individuals requiring insulin therapy and/or to hypoglycaemia-induced falls. Until recently, the inability to account for multiple confounding clinical effects has prevented a clear understanding of the underlying mechanisms for this association. However, we [28] and others [12] have recently shown that insulin treatment remains an independent predictor for fractures, even after type 2 diabetes duration, glucose management and vascular complications are accounted for, and hence the roles of direct insulin effects and falls need to be clarified.

Finally, dramatic weight loss (e.g. following bariatric surgery or a diet very low in energy) is associated with persistent BMD loss, even after weight stabilisation [109, 110]. Negative bone effects appear greatest after malabsorptive procedures [111, 112], highlighting the importance of monitoring bone postoperatively. Understanding whether anti-obesity medications are also associated with BMD loss and increased fractures from weight loss, or, if metabolic remission leads to improvements in skeletal fragility, will be particularly important.

## Conclusions

Skeletal fragility in diabetes is heterogeneous and is associated with a significant clinical burden. Using a bone-centric approach reveals significant gaps in the assessment and management of people with diabetic bone disease. Metabolic dysfunction is associated with, and may contribute to, poorer skeletal outcomes. Incorporating diabetes-specific parameters for skeletal assessment may help to clarify existing inconsistencies, particularly regarding the underlying pathophysiological mechanisms underpinning diabetic osteopathy. Interventional studies of both bone- and metabolic-related treatments with multiple bone endpoints in individuals with well-characterised diabetes could lead to personalised treatment guidelines to improve patient outcomes.

### Supplementary Information

Below is the link to the electronic supplementary material.

Slideset of figures (PPTX 300 KB)

## Abbreviations

aBMD: Areal bone mineral density
AHA: Advanced hip analysis
BMD: Bone mineral density
BTM: Bone turnover marker
DOES: Dubbo Osteoporosis Epidemiology Study
DXA: Dual-energy x-ray absorptiometry
FRAX: Fracture Risk Assessment Tool
HR-pQCT: High-resolution peripheral quantitative computed tomography
SGLT2i: Sodium–glucose cotransporter 2 inhibitors
TBS: Trabecular bone score
VAT: Visceral adipose tissue
vBMD: Volumetric bone mineral density
