# Supplementary material for: Bone metabolism in diabetes: a clinician’s guide to understanding the bone–glucose interplay
Source: Diabetologia. 2024 May 18;67(8):1493–506. doi: 10.1007/s00125-024-06172-x (PMC11343884; doi:10.1007/s00125-024-06172-x)
Supplement: Supplementary file 1 — Slideset of figures (PPTX 300 KB) [file 125_2024_6172_MOESM1_ESM.pptx]

## Slide 1
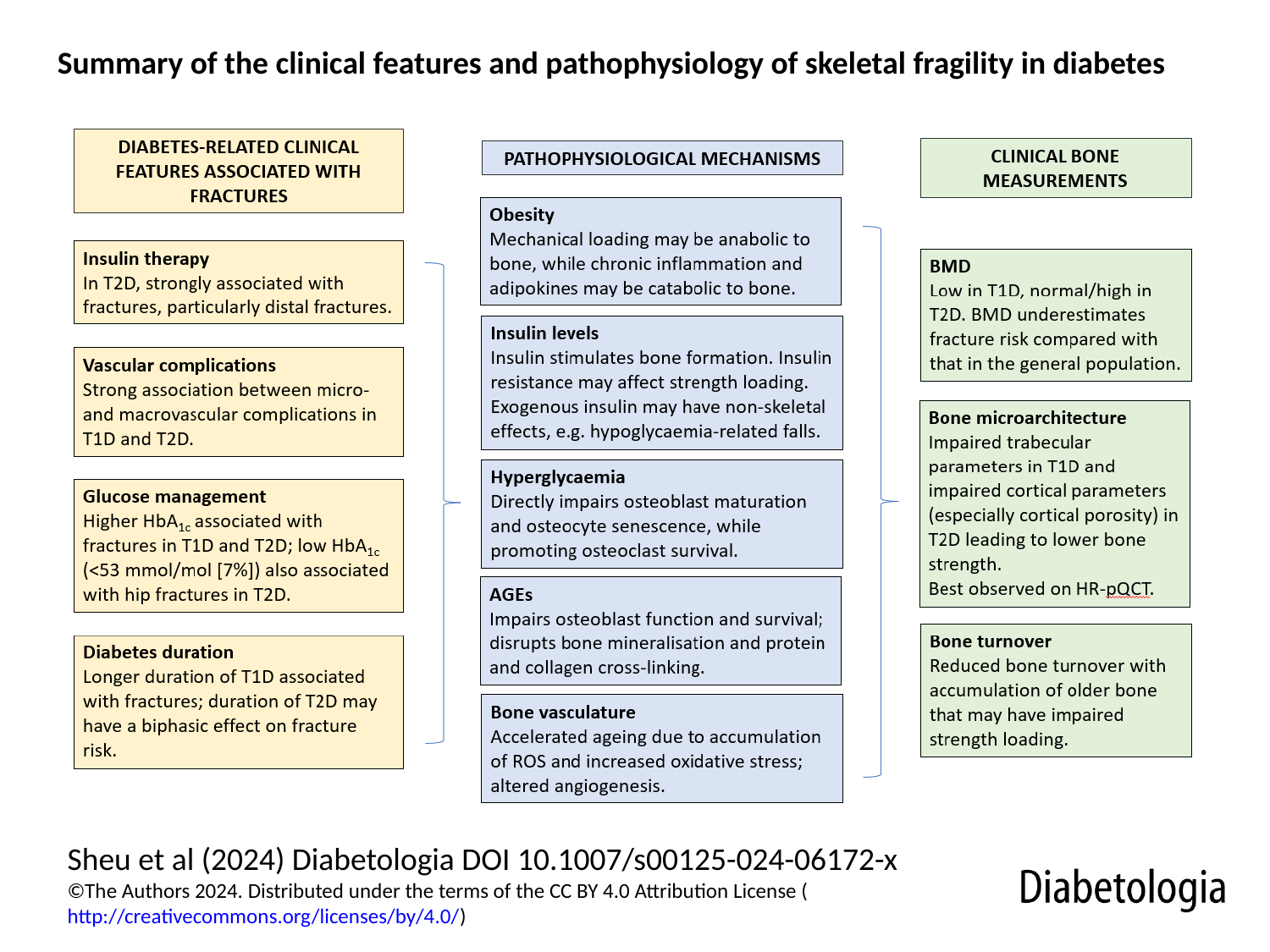

Summary of the clinical features and pathophysiology of skeletal fragility in diabetes
Sheu et al (2024) Diabetologia DOI 10.1007/s00125-024-06172-x
©The Authors 2024. Distributed under the terms of the CC BY 4.0 Attribution License (http://creativecommons.org/licenses/by/4.0/)

## Slide 2
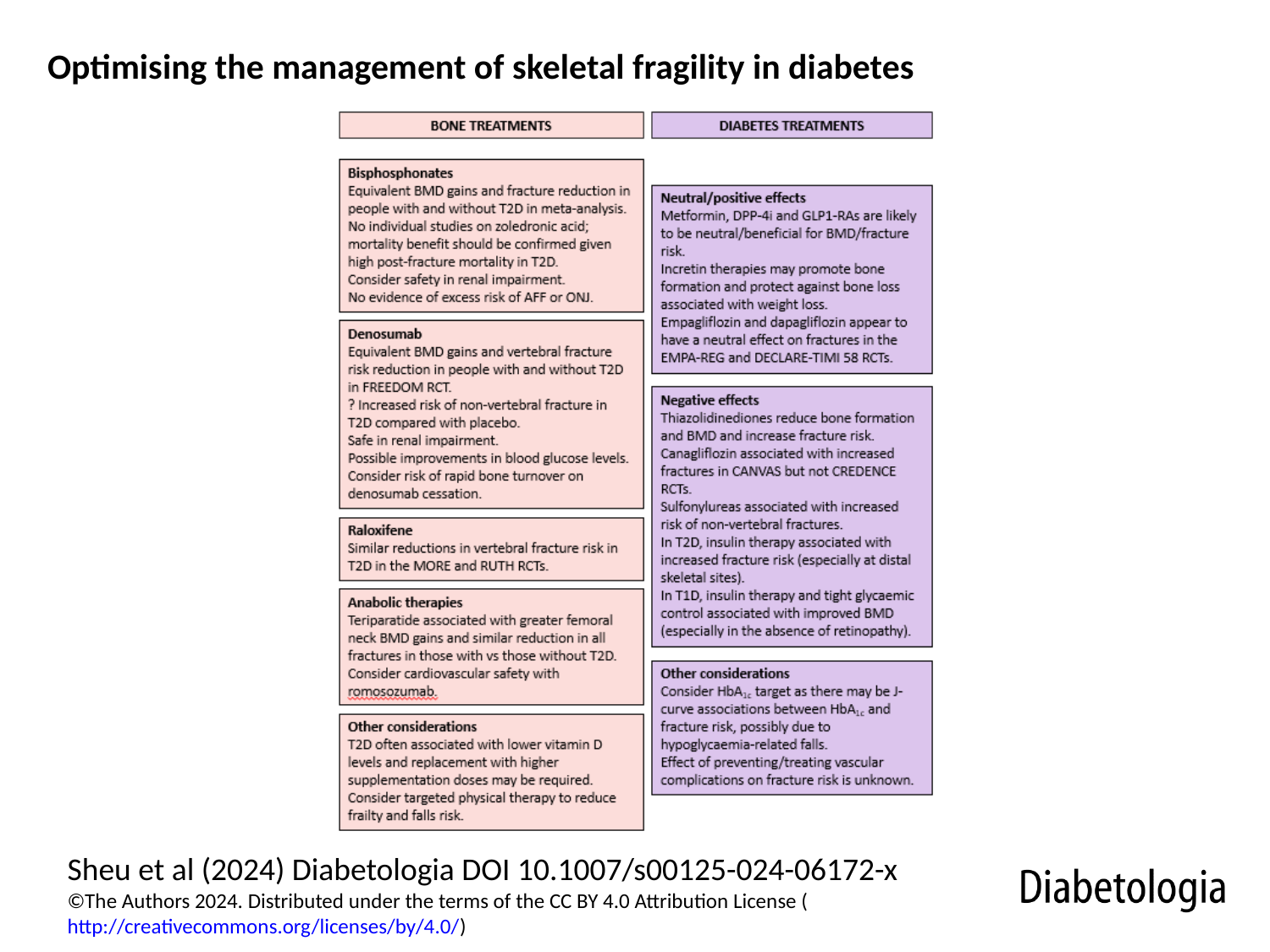

Optimising the management of skeletal fragility in diabetes
Sheu et al (2024) Diabetologia DOI 10.1007/s00125-024-06172-x
©The Authors 2024. Distributed under the terms of the CC BY 4.0 Attribution License (http://creativecommons.org/licenses/by/4.0/)
